# Supplementary material for: Feature analysis of joint motion in paralyzed and non-paralyzed upper limbs while reaching the occiput: A cross-sectional study in patients with mild hemiplegia
Source: PLoS One. 2024 May 23;19(5):e0295101. doi: 10.1371/journal.pone.0295101 (PMC11115294; doi:10.1371/journal.pone.0295101)
Supplement: S1 Table — (DOCX) [file pone.0295101.s001.docx]

**Supporting information**

| **S1 Table. Training methods devised from the pattern analysis results.** | | |
| --- | --- | --- |
| **Reaching motion to the occiput** | | |
| **Training plan** | **Target motor time** | **Training of joint movements** |
| A | Within 1.6 s | Move the joints such that the hand reaches the occiput in the shortest distance and decreases changes in the joint angle. |
| B | Proximity 1.6 s | The change in the angle of flexion of the shoulder and elbow joints increased. |
| C | Proximity 1.6 s | The change in the angle of shoulder abduction is decreased, and the change in the angle of elbow flexion increased. |
| **Reaching motion from the occiput to the starting position** | | |
| **Training Plan** | **Target motor time** | **Training of joint movements** |
| A | Proximity 1.6 s | Let the angular velocity of shoulder abduction and elbow flexion control the angular velocity of elbow flexion. |
