# Supplementary material for: Feature analysis of joint motion in paralyzed and non-paralyzed upper limbs while reaching the occiput: A cross-sectional study in patients with mild hemiplegia
Source: PLoS One. 2024 May 23;19(5):e0295101. doi: 10.1371/journal.pone.0295101 (PMC11115294; doi:10.1371/journal.pone.0295101)
Supplement: Attachment — Submitted filename: 20240502_Response_to_reviewer_plosone.pdf [file pone.0295101.s003.pdf]

Reviewer #3:

Comment 1

I am glad to have the opportunity to review the revised manuscript, which is significantly better than the previous version. However, my main concerns with the revised manuscript are data processing and the statistical tests employed. The authors should address those before publication.

Response to comment 1

We appreciate your insightful comments on the data processing and statistical testing methods in this study. We have referenced your comments and made revisions and additions to the manuscript accordingly.

Comment 2

L4 and L117–119: The comparison of paralyzed and non-paralyzed upper limbs in this study design is a limitation despite being a clinically focused point. Mentioning this in the title and the study objective will help your readership to focus on the target audience.

Response to comment 2

We appreciate your helpful suggestions. We have revised our title and research objectives.

"Feature analysis of joint motion in paralyzed and non-paralyzed upper limbs while reaching the occiput: a cross-sectional study in patients with mild hemiplegia" (Title, lines 4–5)

"This cross-sectional study aimed to clarify in patients with mild hemiplegia the kinematic characteristics <u>of paralyzed and non-paralyzed upper limbs</u> reaching the occiput." (Abstract, lines 29–31)

"Based on the above discussion, this study aimed to clarify the kinematic characteristics <u>of paralyzed and non-paralyzed upper limbs</u> in patients with chronic phase mild hemiplegia while reaching the occipital region with the hand using 3DMA." (Introduction, lines 119–121)

Comment 3

L158–161, P7: In G*power, I find this only in the exact test, and the effect size "g" is displayed there. What test family was used in your calculation? Also, what was your required sample size?

Response to comment 3

We appreciate your remarks regarding sample size. We have corrected an error in our description of how we calculated the sample size using G*power. For the G*power calculation, the selected test family was “Exact”, and the sample size was calculated to be eight patients. The results of the sample size calculation are presented in the screenshot below.

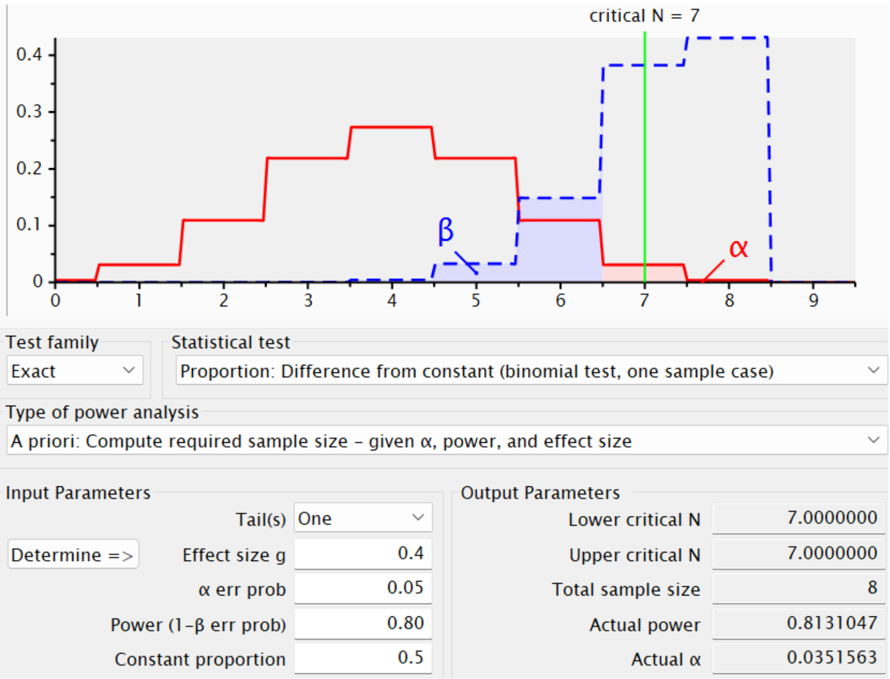

“The sample size was calculated by setting the difference from the constant (test family: exact test, binomial test, one sample case). For calculating the required sample size, the effect size (g) was 0.4, α was 0.05, power (1-β) was 0.8, and the constant proportion was 0.5.” (Materials and methods, Sample size, lines 161–164)

Comment 4

L185, P8: In this figure caption, please explain the landmark.

Response to comment 4

We appreciate your suggestion. We have added a description of the placed landmark to the caption of Fig 2.

“**Fig 2. Measurement environment.** (a) Side view and (b) top view. A landmark was placed 5 m away from the participants. By looking at it, neck and trunk movement was minimized in participants

performing the reaching task." (Materials and methods, Experimental procedure, lines 188–190)

Comment 5

L223, P9: For illustrations in each trial, left is outward, and right is return motions. This should be added to the figure caption.

Response to comment 5

We refer to your comments and have added an explanation of the illustration to the caption of Fig 4.

"**Fig 4. Measurement sequence**. The illustrations on the left of each trial show the outward motion from the starting position until the hand reaches the back of the head, and the illustrations on the right show the return motion from the point where the hand reaches the back of the head to the original position." (Materials and methods, Reaching task, lines 229–232)

Comment 6

L246–251, P10–11: Did you input 50 data points into descriptive statistics and statistical tests? In general, each participant's data is averaged first, then descriptive statistics are calculated, after which statistical tests are employed. In general, averaging trial data for each participant is performed to correct measurement errors. This is followed by statistical tests, which are performed to estimate the population.

Response to comment 6

Thank you for your valuable feedback on our statistical approach regarding the analysis of data points in our manuscript. In our analysis, descriptive statistics, multivariate analysis of covariance (MANCOVA), and random forest analyses were performed on 50 data (10 patients × 5 trials) for each paralyzed and non-paralyzed side. We did not analyze the data as 10 data averaged over 5 trials per person. We understand your concern about the typical process of averaging individual participant data before conducting further statistical analyses. This method is indeed a common approach to minimize variability due to measurement errors and to ensure that the analysis reflects the central tendency of each participant's performance.

In our study, the approach to analyzing individual data points without first averaging them was intentional and is justified by the following considerations:

1. Individual Variation Exploration: Our study aimed not only to explore general trends but also to capture individual variations in motor performance across trials. This was particularly important as we

hypothesized that there might be significant trial-to-trial variability in motor task performance among stroke survivors, which could be clinically relevant.

2. Data Structure and Analysis Technique: We input 50 data points directly into the descriptive and inferential statistical analyses to maintain the richness of the data, which includes within-subject variability. This approach allowed us to apply more complex models that consider both within-subject (repeated measures) and between-subject variability simultaneously, enhancing the robustness of our findings.

3. Statistical Rigor: We employed statistical techniques that are robust to the inclusion of multiple measurements from the same participants, such as MANCOVA and mixed-effects models, which inherently adjust for the non-independence of repeated measurements within subjects.

However, we acknowledge that averaging data prior to analysis is a common practice and can be beneficial in simplifying the data presentation and interpretation. We have added the following explanation of data averaging to the manuscript.

“Data from the same participant were not averaged to allow subsequent analyses using random forest clustering.” (Materials and methods, Data acquisition and analysis, lines 260–261)

Comment 7

L301, P13: Provide the effect size calculation and eligibility criteria.

Response to comment 7

Our description of the effect size calculation was indeed missing. Based on your comments, we have added the following statement to the manuscript.

“The η^2 was used for the effect size in the statistical analysis, and the effect size indices were set as 0.01 for small, 0.06 for medium, and 0.14 for large [46].” (Materials and methods, Statistical analysis, lines 318–320)

“Reference #46 Cohen J. Statistical power analysis for the behavioral sciences. 2nd ed. New Jersey: L. Erlbaum Associates; 1988.”

Comment 8

L307, P13: To my knowledge, MANCOVA is used to compare independent samples, which means it is not applicable here.

Response to comment 8

Thank you for your constructive comment regarding the MANCOVA use in our study. We acknowledge the concern that MANCOVA is typically used for comparing independent samples. In our study, we employed MANCOVA to assess differences in joint motion characteristics between paralyzed and non-paralyzed limbs within subjects who have undergone stroke rehabilitation. This method was chosen to control for potential confounding variables such as age, sex, and body mass index, which might influence the motor outcomes. The following recently published papers use the MANCOVA (within-subjects design) analysis with repeated measures as in the present study.

Thompson CM, Voorhees HL, Taniguchi-Dorios E, Makos S, Pool K, Babu S. Development and initial assessment of an emotional support provision training intervention for interpersonal support providers in the context of chronic illness. Health Commun. 2024 Mar 11:1-14. doi: 10.1080/10410236.2024.2325183.

Figueroa-Padilla I, Rivera Fernández DE, Cházaro Rocha EF, Eugenio Gutiérrez AL, Jáuregui-Renaud K. Body weight may have a role on neuropathy and mobility after moderate to severe COVID-19: an exploratory study. Medicina (Kaunas). 2022 Oct 6;58(10):1401. doi: 10.3390/medicina58101401.

We recognize that the use of MANCOVA in a repeated measures context (within-subject design) is less typical and might have led to confusion. Our intention was to exploit the multivariate capability of MANCOVA to handle multiple dependent variables—motor time, joint angles, and angular velocities—which are not independent of each other.

We appreciate the opportunity to improve the clarity and accuracy of our analysis, and we have revised our manuscript to better justify our choice of statistical methods or adapt our approach in line with best statistical practices.

“Repeated measures multivariate analysis of covariance was performed on these data.” (Abstract, line 36)

“A repeated measures multivariate analysis of covariance (within-subject design) was performed to test this hypothesis.” (Materials and methods, Statistical analysis, lines 313–314)

“Binomial logistic regression analysis was conducted on the features fitted to the model for the paralyzed and non-paralyzed sides using repeated measures multivariate analysis of covariance.” (Materials and methods, Statistical analysis, lines 322–324)

“Repeated measures multivariate analysis of covariance was performed.” (Results, Detection of paralyzed upper limb with motion data, line 412)

“Binomial logistic regression analysis of the model-fitted features on the paralyzed and non-paralyzed sides was performed using repeated measures multivariate analysis of covariance.” (Results, Detection of paralyzed upper limb with motion data, lines 429–431)

“A repeated measures multivariate analysis of covariance was performed to test the hypothesis that motor time, joint angles, and angular velocity while doing movements to reach the occiput differ between paralyzed and non-paralyzed upper limbs.” (Discussion, lines 517–519)

“Next, to detect the cutoff values of the motor features that discriminate between the paralyzed and non-paralyzed sides, binomial logistic regression analysis was conducted on the features fitted to the models for the paralyzed and non-paralyzed sides using repeated measures multivariate analysis of covariance.” (Discussion, lines 525–528)

We appreciate all the comments and suggestions provided in order to improve our manuscript, and we sincerely thank all the reviewers.
