# Supplementary material for: Feature analysis of joint motion in paralyzed and non-paralyzed upper limbs while reaching the occiput: A cross-sectional study in patients with mild hemiplegia
Source: PLoS One. 2024 May 23;19(5):e0295101. doi: 10.1371/journal.pone.0295101 (PMC11115294; doi:10.1371/journal.pone.0295101)
Supplement: Attachment — Submitted filename: 20240313_Response to reviewers_plosone.docx [file pone.0295101.s002.docx]

**Journal requirements**

**Comment 1**

**Please ensure that your manuscript meets PLOS ONE's style requirements, including those for file naming. The PLOS ONE style templates can be found at**

[**https://journals.plos.org/plosone/s/file?id=wjVg/PLOSOne_formatting_sample_main_body.pdf**](https://journals.plos.org/plosone/s/file?id=wjVg/PLOSOne_formatting_sample_main_body.pdf)

[**https://journals.plos.org/plosone/s/file?id=ba62/PLOSOne_formatting_sample_title_authors_affiliations.pdf**](https://journals.plos.org/plosone/s/file?id=ba62/PLOSOne_formatting_sample_title_authors_affiliations.pdf)**.**

Response to comment 1

We have verified that the manuscript meets all the style requirements of PLOS ONE. We also confirm that the manuscript has been edited by Editage (<https://www.editage.jp/>) and follows the specified guidelines.

**Comment 2**

**We note that you have indicated that there are restrictions to data sharing for this study. PLOS only allows data to be available upon request if there are legal or ethical restrictions on sharing data publicly. For more information on unacceptable data access restrictions, please see** [**http://journals.plos.org/plosone/s/data-availability#loc-unacceptable-data-access-restrictions**](http://journals.plos.org/plosone/s/data-availability#loc-unacceptable-data-access-restrictions)**.**

**Before we proceed with your manuscript, please address the following prompts:**

**a) If there are ethical or legal restrictions on sharing a de-identified data set, please explain them in detail (e.g., data contain potentially identifying or sensitive patient information, data are owned by a third-party organization, etc.) and who has imposed them (e.g., a Research Ethics Committee or Institutional Review Board, etc.). Please also provide contact information for a data access committee, ethics committee, or other institutional body to which data requests may be sent.**

**b) If there are no restrictions, please upload the minimal anonymized data set necessary to replicate your study findings to a stable, public repository and provide us with the relevant URLs, DOIs, or accession numbers. For a list of recommended repositories, please see**

**https://journals.plos.org/plosone/s/recommended-repositories. You also have the option of uploading the data as Supporting Information files, but we would recommend depositing data directly to a data repository if possible.**

Response to comment 2

Uploading this study's dataset to public repositories is restricted by the ethics committee that approved this study. This data contain potentially identifying or sensitive patient information; therefore, researchers who wish to use this dataset may obtain it after submitting an individual application to the following ethics committee:

The Jikei University Institutional Review Board,

3-25-8 Nishi-Shimbashi, Minato-ku, Tokyo, 105-8461

Tel: +81-3-3433-1111 Ext 2187

Fax: +81-3-5400-1388

Email: [rinri@jikei.ac.jp](mailto:rinri@jikei.ac.jp)

**Reviewer #1**

**The article “Feature analysis of joint motion while reaching the occiput in patients with mild hemiplegia: a cross-sectional study” aims at better characterizing motor strategy in post-stroke patients during one peculiar task, i.e. reaching the back of the head. The protocol is well described and the data analysis method proposed is interesting to go deeper into the understanding.**

**Comment 1**

**My main concern is that the goal is too fuzzy as the rationale is too light. It is mainly saying: there are too few articles providing raw data regarding that particular task (reaching the back of the head with one hand) so we will perform 3D measures and provide an analysis of raw data. Why this task rather than another? Several tasks implying to bring the hand in the posterior side of the body might be as important as this one, e.g. mid-back or low-back. What is useful for to better know the specific characteristics of post-stroke people motor strategy to achieve this particular task? I am not saying that is useless, only that you do not explain clearly why it is useful in a clinical perspective.**

Response to comment 1

Thank you for your important comments. We have added the following explanation regarding the significance of this study.

“Reaching the back of the head requires a wide range of motion and coordination between the shoulder and elbow joints, which is a challenging task for patients with motor paralysis. Besides the occiput, the mouth, chest, abdomen, and lower back are other reaching target points for eating, dressing, and bathing. While important for self-care performance, reaching these target points is less difficult than reaching to occipital area, thus, patients with mild hemiplegia are often able to perform these reaches adequately [7,8]. The ability to smoothly reach the back of the head, which is difficult for hemiplegics, is beneficial to their ADLs.” (Introduction, lines 62–69)

“Due to a ceiling effect, upper extremity function scores used in clinical practice do not measure the recovery in patients with mild motor paralysis [14,15]. When planning a change in joint movement pattern exercises, or to shorten their duration, attempting to use the clinically obtained scores is challenging. To the best of our knowledge, a kinematic index that quantitatively expresses the "reach to the back of the head" does not exist. Moreover, a clear target value to determine if the reach has been sufficiently recovered in patients with mild motor paralysis is lacking.” (Introduction, lines 81–87)

**Comment 2**

**L50: “to improve motor paralysis” sounds a bit strange. It should rather be “to improve motor capacities”**

Response to comment 2

We have revised the text as follows.

“As motor paralysis of the upper limbs and fingers limits patients' activities of daily living (ADLs) and decreases their quality of life (QOL), patients are provided with continuous rehabilitation to improve their motor capacities [3,4].” (Introduction, lines 52–54)

**Comment 3**

**L52: “Reaching is a fundamental movement of the upper limb, with the goal of reaching the target point” please rephrase. It is a loop; you define “reaching” by “reaching”**

Response to comment 3

We have made modifications to the definition of Reaching.

“Reaching is a basic movement of the upper extremity aimed at extending the arm toward a designated target in order to touch or grasp something [5]. ” (Introduction, lines 55–57)

**Comment 4**

**L81: “because of the discrepancy between the results and the upper limb movements in daily activities” I do not understand what could be a discrepancy between results (which ones?) and movements. Do you mean movements performed in the protocol are too far from ADL? Or do you mean the results of several studies are not consistent? The link with the next sentence, “As the tasks are measured in a manner that is close to daily life situations…”, is also not clear. You’ve just said that they are too far from properly represent ADL and then you write they are close. Is it far or close?**

Response to comment 4

We apologize for the misunderstanding. We did not want to emphasize the discrepancies between studies. We wished to highlight the usefulness of measuring movements that are similar to ADLs. We have revised the text in this regard.

“Kinematic characteristics calculated by analyzing movements related to daily life are strongly related to the patient's activity capacity, and are valid indicators of patient treatment in clinical practice [21–24].” (Introduction, lines 93-96)

**Comment 5**

**L85: “the kinematic characteristics of patients with stroke with mild motor paralysis have not been verified” What do you mean by “verified”? We verify hypotheses but how can we verify characteristics?**

Response to comment 5

We have not explained this point well enough. We have added the following text.

“Several studies have evaluated healthy participants and patients with orthopedic diseases in terms of their ability to reach the occipital region; however, kinematic characteristics such as motor time, angular change of joints, and angular velocity of joints have not been validated in stroke patients with mild motor paralysis [9,10,25]. This issue can be analyzed using 3DMA to provide quantified data.” (Introduction section, lines 96–100)

**Comment 6**

**L90: “When upper limb motor tasks are measured using a three-dimensional motion analyzer, the motions of the paralyzed and non-paralyzed sides should be compared” I do not agree with this. The reference for one given motion of paretic limb is not always the corresponding motion of non-paretic limb. Healthy people have obviously asymmetric motor strategies for some many tasks. Comparing dominant and non-dominant limbs’ motions is not the quantification of the effect of a deficiency. Your sentence is only true if you have previously demonstrated that, for one given task, motor strategy is symmetric in healthy population, meaning intra-subject variability (left w.r.t right) is lower than inter-subjects’ one.**

Response to comment 6

Thank you for your helpful remarks on this important aspect of our study. We should have been careful in our discussion about comparing the paralyzed side with the non-paralyzed side. We have revised the manuscript and added the following text in response to your comments.

“Upper extremity motor tasks can be analyzed using 3DMA by comparing healthy subjects and patients; however, in clinical situations, movements on the paralyzed and nonparalyzed side of the patient may be compared [26]. The movements of the nonparalytic upper limb in patients with hemiplegia after a stroke differ from those of normal participants, and the reference for a paralyzed movement is not necessarily the corresponding movement of the nonparalyzed limb. Although the nonparalyzed upper limb may also have compensatory strategies for movement, a comparison of the kinematic characteristics of the paralyzed and nonparalyzed upper limbs of patients with hemiplegia with mild impairment of limb and trunk function has been conducted to validate the calculation of the evaluation values that will be used as a reference for treatment [27,28].” (Introduction, lines 103–112)

“Third, in the present analysis, it was not proven that the within-subject variability (left-right difference) was smaller than the between-subject variability. Because normal subjects have clearly asymmetric motor strategies in many movements, the criterion for a paralyzed movement is not necessarily the corresponding movement of the nonparalyzed limb. To resolve this limitation, the present upper limb task should be analyzed in the movements of healthy subjects to verify that it is symmetrical [66].” (Discussion, lines 620–626)

**Comment 7**

**L92: “however, the non-paralytic upper limb, which does not show obvious functional impairment, can be used in clinical situations for comparison with the paralytic upper limb” I do not fully agree with this sentence either. Compensation strategies in non-paretic limb are neglected.**

Response to comment 7

Thank you for pointing this out. The limitations of comparing the paralyzed and non-paralyzed sides in this study should have been clearly stated. We have added the following text along with the response to your Comment 6.

“Third, in the present analysis, it was not proven that the within-subject variability (left-right difference) was smaller than the between-subject variability. Because normal subjects have clearly asymmetric motor strategies in many movements, the criterion for a paralyzed movement is not necessarily the corresponding movement of the nonparalyzed limb. To resolve this limitation, the present upper limb task should be analyzed in the movements of healthy subjects to verify that it is symmetrical [66].” (Discussion, lines 620–626)

**Comment 8**

**L111: “while reaching the hand to the back of the head” is not clear. Patient are not reaching the hand but reaching the back of their head with their hand.**

Response to comment 8

We have refered to your comments and modified the text as follows.

“In this cross-sectional study, kinematic data of patients with post-stroke hemiplegia were obtained while reaching the back of their head with their hand, and the patient’s paralyzed and non-paralyzed upper limbs were compared.” (Materials and methods, Study design, lines 126–128)

**Comment 9**

**P128: Rather than exclusion criteria, I would recommend to use non-inclusion criteria. To me exclusion criteria stand for situations occurring after inclusion that force experimenters to withdraw one participant from the study while he/she has already started the experiment. On the opposite, non-inclusion means that this person has never been a participant, i.e. has never started the experiment.**

Response to comment 9

Thank you for your important comments. We have decided to use the phrase "non-inclusion criteria" in the text revised the text accordingly.

“The non-inclusion criteria were as follows: cases with a paralyzed hand not reaching the external occipital ridge with automatic movements; presence of a central nervous system disease other than stroke, orthopedic disease, mental disorder, cognitive impairment after stroke, dementia, visual field disorder, and ataxia at diagnosis; subluxation of the shoulder joint; pain in the joints of the upper limb or fingers during exercise; presence of a significant limitation in the joint range of motion in the upper limb; and completion of the occupational therapy intervention. Patients who met the study eligibility criteria but did not meet the non-inclusion criteria were asked to participate in the study, and those who provided consent were considered participants.” (Materials and methods, Participants, lines 143–151)

**Comment 10**

**L152 and following: what is the need for markers on lower limbs if participants remain seated and if you are only analyzing upper body joints motions?**

Response to comment 10

As you pointed out, there is a model in which markers are placed only on the upper limbs. However, owing to the version of our analysis equipment, it was difficult to perform the analysis using such a model. Therefore, the Plug-in Gait model, one of the commonly used models, was selected for this study. The choice of this model did not impose significantly more time constraints on the participants than the upper extremity model, because the examiners were skilled at applying the markers.

**Comment 11**

**L161: what do you mean by “thermal cameras”? Is it infrared cameras?**

Response to comment 11

Yes, we used infrared cameras in this study. We have modified the text as follows.

“Six infrared cameras were placed on the ceiling of the room, and a landmark was placed 5 m away from the participants (Fig 2).” (Materials and methods, Experimental procedure, lines 178–179)

**Comment 12**

**L161: what is the goal of the “landmark placed 5m away from the participants”? On L188 you explain that participants had to maintain gaze on this landmark.**

Response to comment 12

As you mention, we did not describe the purpose of the landmark. We have added a sentence in this regard.

“The landmark was placed to minimize neck and trunk movement while the participants performed the reaching task looking at it.” (Materials and methods, Experimental procedure, lines 179–180)

**Comment 13**

**L179: “elbow in full extension, forearm in mid-extension” This is a bit strange. Once you are speaking about a joint then about a segment. By forearm, did you actually mean wrist? Why was it in mid-extension rather than in neutral posture? Furthermore, was this posture (elbow extension, wrist mid-extension, …) always reachable for patients, especially with paretic limb? Considering the usual triple flexion posture observed in that limb, it seems that the starting position you ask is hardly reachable. Your following sentence, i.e. relax the muscles as much as possible, is indeed a more valuable instruction. Fig 2b does not illustrate at all this instruction. L195, you precise “the examiner verified whether the participants were in the correct starting position”. This is a bit contradictory with sentence on L181-183. What was the actual tolerance for the starting position? L179: "elbow in full extension, forearm in mid-extension"**

Response to comment 13

Thank you very much for your important remarks. There was an error in the English wording in this sentence. The forearm was not mid-extension, but mid-position between supination and pronation. We have corrected the sentence as follows.

“The starting position of the upper limb on the measurement side was set with the elbow in full extension, fingers in extension, and the forearm in mid-position between supination and pronation; the forearm and fingers were not in contact with the chair.” (Materials and methods, Experimental procedure, lines 168–171)

The patients with mild motor paralysis who participated in this study were able to achieve the starting limb position easily in the first half of the measurement because only two patients had moderate spasticity, and none of them had significant joint contractures. Eight patients had a mAs of 1 and 2 had a mAs of 1+ in the biceps muscle. However, as you pointed out, some of them could not fully reach the starting position because of an increased muscle tone caused by the repeated measurements. In this respect, the participants were allowed to relax their muscles during the one-minute rest period to reproduce the starting limb position as much as possible. After the break was over, we allowed some participants to be unable to reach the starting position, for example, with a slight flexion of the elbow joint. We modified the text in this regard as follows.

“Before each measurement, the examiner instructed the participants to attempt to achieve the starting position with the upper limb as much as possible. If a participant was unable to assume the starting position due to heightened muscle tone in their upper limb, causing a slight bending of the elbow joint, the procedure was still considered appropriate.” (Materials and methods, Reaching task, lines 215–218)

We added a Figure demonstrating the starting limb position during the measurement of the task, and the Figure that showed the measurement environment was cited in the text accordingly.

“The upper limb on the non-tested side was also placed in the same position (Fig 1).” (Materials and methods, Experimental procedure, lines 171–172)

“Fig 1. Starting position during the measurement. (a) side view of the starting position and (b) starting position of the upper limb.” (Materials and methods, Reaching task, lines 182–183)

“Six infrared cameras were placed on the ceiling of the room, and a landmark was placed 5 m away from the participants (Fig 2).” (Materials and methods, Reaching task, lines 178–179)

“Fig 2. Measurement environment. (a) side view and (b) top view.” (Materials and methods, Reaching task, line 185)

**Comment 14**

**L279: “to test the hypothesis that the kinematic characteristics […] differ between the paralyzed and non-paralyzed upper limbs”. First, this hypothesis must be better explained in introduction section. Then, this hypothesis is much too large. Even if you specify just after the independent variables taken into account, such a large hypothesis allows that at least one parameter will differ. Finally, and once again, a difference between paretic and non-paretic limbs in this population only means something under the hypothesis that the corresponding difference (dominant vs. non-dominant) does not exist in healthy population.** **L279: 「to test the hypothesis that the kinematic characteristics [...] differ between the paralyzed and nonparalyzed upper limbs"**

Response to comment 14

We have explained the study's hypothesis by providing a detailed description of how the paralyzed and nonparalyzed sides were compared.. We also added in the study limitations that we compared the patient's movements on the paralyzed side to those on the nonparalyzed side. We modified our description of the study's hypothesis and the limitations as follows.

“Assuming that there is no left-right difference in the movement pattern between the dominant and nondominant upper limbs for reaching the back of the head with the hand in individuals without motor paralysis, we hypothesized that movement time, joint angles, and angular velocity while performing the movement of reaching the back of the head with the hand in patients with chronic phase mild hemiplegia will differ between the paralyzed and non-paralyzed upper limbs. A multivariate analysis of covariance was performed to test this hypothesis.” (Materials and methods, Statistical analysis, lines 302–308)

“In this study, assuming that there is no left-right difference in the movement pattern between the dominant and non-dominant upper limbs for reaching the back of the head with the hand in individuals without motor paralysis, we hypothesized that movement time, joint angles, and angular velocity while performing the movement of reaching the back of the head with the hand in patients chronic phase with mild hemiplegia will differ between the paralyzed and non-paralyzed upper limbs. A multivariate analysis of covariance was performed to test the hypothesis that motor time, joint angles, and angular velocity while doing movements to reach the occiput differ between paralyzed and non-paralyzed upper limbs.” (Discussion, lines 506–511)

“Third, in the present analysis, it was not proven that the within-subject variability (left-right difference) was smaller than the between-subject variability. Because normal subjects have clearly asymmetric motor strategies in many movements, the criterion for a paralyzed movement is not necessarily the corresponding movement of the nonparalyzed limb. To resolve this limitation, the present upper limb task should be analyzed in the movements of healthy subjects to verify that it is symmetrical [66].” (Discussion, lines 620–626)

**Comment 15**

**L310: what is IG in equation 2? Moreover, this equation could be re-written considering paretic/non-paretic limbs rather than left/right.**

Response to comment 15

We have added the following explanation for IG and modified the text as follows.

“To determine which features to use as data partition nodes in Equation 1, information gain was measured using a Random Forest in Equation 2. The information gain is calculated for all the possible split points (each feature's value) in the data set, and the feature f with its split point with the largest information gain were selected. Random forests are characterized by the random selection of a subset of features to be used in the construction of each decision tree. The process of selecting the feature f that maximizes the information gain in Equation 2 from the features in Equation 1 data ensures model diversity, suppresses overlearning, and improves generalization performance.” (Materials and methods, Statistical analysis, lines 334–341)

“In equation 1, *x* is the feature value of the paralyzed side, *y* is the value of the paralyzed side as the objective variable, and *k* is the total number of features. In Equation 2, *IG* is the information gain in a random forest, which is the sum of the impurity of the left and right child nodes from the impurity of the parent node, weighted by their ratio; *D_p_* is the parent dataset; *f* is the number of explanatory variables; *N* is the depth of the decision tree; *D_non-paretic_* is the non-paretic child node from the parent node; *D_paretic_* is the paretic child node from the parent node; and I is the impurity.” (Materials and methods, Statistical analysis, lines 346–352)

We have modified Equation 2 in reference to your comment.

$IG\left( D_{p}, f \right)=I\left( D_{p} \right)-\frac{N_{non-paretic}}{N_{p}}I\left( D_{non-paretic} \right)-\frac{N_{paretic}}{N_{p}}I\left( D_{paretic} \right)$ …(Equation 2)

(Materials and methods, Statistical analysis, line 344)

**Comment 16**

**L332: “Of the 88 patients, 75 were excluded” How this is possible? What is the reason for such a discrepancy between first screening and inclusion? Were the non-included patients particularly affected or was the FMA or ARAT threshold too high?**

Response to comment 16

Participants were excluded because most of the patients who attended the study facilities had severe hemiplegia. The current study included patients with mild hemiplegia who were able to reach the back of their heads with their hands. As you pointed out, 60 patients were excluded because they had low FMA and ARAT scores and could not reach the back of their heads with their hands.

**Comment 17**

**L341: “All the participants had right hemiplegia” Was it a coincidence or is there something in the inclusion/non-inclusion criteria that favor inclusion of right hemiplegia? It is a bit surprising as they were also all right-dominant before stroke and therefore potentially more affected by hemiplegia.**

Response to comment 17

Thank you for pointing this out. The inclusion/non-inclusion criteria in this study did not specify the paralytic or dominant side of the participants. All the participants in the final analysis had right hemiplegia and were right-handed before the stroke, which happened by chance and not because of intentional adjustement. We have described this as a limitation of our study as follows.

“Third, in the present analysis, it was not proven that the within-subject variability (left-right difference) was smaller than the between-subject variability. Because normal subjects have clearly asymmetric motor strategies in many movements, the criterion for a paralyzed movement is not necessarily the corresponding movement of the nonparalyzed limb. To resolve this limitation, the present upper limb task should be analyzed in the movements of healthy subjects to verify that it is symmetrical [66].” (Discussion, lines 620–626)

Comment 18

**Table 2: variability in peak angular velocities is very high. Did you check normality for these parameters before comparing sides?**

Response to comment 18

Thank you for pointing this out. We confirmed that the angular velocity data were not normally distributed by the Shapiro-Wilk test. The manuscript lacked an explanation of the analysis method for variables that are not normally distributed. We have added this point to the manuscript.

“Variables for which the Shapiro-Wilk test confirmed that the data were not normally distributed were compared using the Kruskal-Wallis test.” (Materials and methods, Statistical analysis, lines 311–313)

**Comment 19**

**Figures are hardly readable**

Response to comment 19

Thank you very much for your suggestion. We have increased the font size in the Figures.

**Reviewer #2:**

**The manuscript is well written and I identify a major conercern that deserves to be strenghten. In my opinion is very important to stress the rationale bheind the study and the application that a study with a strong kinesiological background may have in relation to the development of modern assistive technologies. For this reason I suggest the Authros to betterframe this aspect both in the introduction and in the discussion. Under this perspective I suggest Authros to rviewe the following recent manuscripts:**

**-"On the Decoding of Shoulder Joint Intent of Motion from Transient EMG: Feature Evaluation and Classification." IEEE Transactions on Medical Robotics and Bionics (2023)".**

**-"Rehabilitation with Kinematic Biofeedback Improves Shoulder Function in Patients Surgically Treated for Rotator Cuff Tear: Indications from a Randomized Controlled Trial." (2023).**

Response to comment

Thank you very much for reviewing our manuscript. We have read the papers you provided and revised the Introduction and Discussion. We have referred to your comments and explained the significance of this study and the application of the results.

“Kinematic quantification of movement is strongly recommended to distinguish between the recovery of motor strategies and improvement owing to motor compensations; therefore, it is increasingly used in clinical practice as a new outcome measure [19,20]. Kinematic characteristics calculated by analyzing movements related to daily life are strongly related to the patient's activity capacity, and are valid indicators of patient treatment in clinical practice [21–24]. Several studies have evaluated healthy participants and patients with orthopedic diseases regarding their ability to reach the occipital region; however, kinematic characteristics such as motor time, angular change of joints, and angular velocity of joints have not been validated in stroke patients with mild motor paralysis [9,10,25]. This issue can be analyzed using 3DMA to provide quantified data.” (Intoroduction, lines 91–100)

“Reference # 19 Kwakkel G, Lannin NA, Borschmann K, English C, Ali M, Churilov L, Saposnik G, Winstein C, van Wegen EEH, Wolf SL, Krakauer JW, Bernhardt J: Standardized Measurement of Sensorimotor Recovery in Stroke Trials: Consensus-Based Core Recommendations from the Stroke Recovery and Rehabilitation Roundtable. Neurorehabil Neural Repair 2017;31:784-792.

“Reference #20 Demers M, Levin MF: Do Activity Level Outcome Measures Commonly Used in Neurological Practice Assess Upper-Limb Movement Quality? Neurorehabil Neural Repair 2017;31:623-637.”

”

“Reference #21 Alt Murphy M, Willén C, Sunnerhagen KS: Movement kinematics during a drinking task are associated with the activity capacity level after stroke. Neurorehabil Neural Repair 2012;26:1106-1115.

”

“In addition, clustering patterns of movement time and joint angle changes from the data obtained using opportunity learning would be useful for providing appropriate exercises for patients [29].” (Introduction, lines 114–116)

“Reference #29 Formstone L, Huo W, Wilson S, McGregor A, Bentley P, Vaidyanathan R. Quantification of motor function post-stroke using novel combination of wearable inertial and mechanomyographic sensors. IEEE Trans Neural Syst Rehabil Eng. 2021;29:1158-67. Epub 20210624. doi: 10.1109/tnsre.2021.3089613.

“The quality of upper extremity movement can be improved by exercises performed based on kinematic values [51]. Recently, kinematic biofeedback has been developed as a new rehabilitation therapy [52]. This is a treatment in which patients are given exercises while alternative proprioceptive feedback is returned to them based on the results measured by 3DMA and electromyography. Although biofeedback has been introduced to restore the function of reaching movements in stroke patients, the effectiveness of the treatment has not been fully tested, and solid recommendations have not been provided [53]. The results of this study are useful as reference data for the application of biofeedback, robotics rehabilitation, and other technologies, and are expected to contribute to the development of therapies for restoring the patients' functions and activity limitations.” (Discussion, lines 529–538)

“Reference #51 Prange-Lasonder GB, Alt Murphy M, Lamers I, Hughes AM, Buurke JH, Feys P, Keller T, Klamroth-Marganska V, Tarkka IM, Timmermans A, Burridge JH: European evidence-based recommendations for clinical assessment of upper limb in neurorehabilitation (CAULIN): data synthesis from systematic reviews, clinical practice guidelines and expert consensus. J Neuroeng Rehabil. 2021;18:162.”

“Reference #52 Giggins OM, Persson UM, Caulfield B: Biofeedback in rehabilitation. J Neuroeng Rehabil 2013;10:60.”

“Reference #53 Pelton T, van Vliet P, Hollands K: Interventions for improving coordination of reach to grasp following stroke: a systematic review. Int J Evid Based Healthc 2012;10:89-102.”

**Reviewer #3:**

**Comment 1**

**This manuscript suggests kinematic values for reaching the occiput, aiming to enhance patients' daily movements. This approach holds promise for upper-limb kinematic analysis in clinical settings. However, several concerns need addressing before publication. Primarily, redundancies in the descriptions have been identified. It is advisable to review the use of abbreviations and incorporate subheadings within the Materials and Methods section. Additional comments are outlined below:**

Response to comment 1

We are very grateful to you for your thoughtful and valuable comments on this manuscript. We have defined abbreviations, revised section subheadings, and made corrections and additions to the text based on your comments.

**Comment 2**

**Abstract**

**L 26, P 2: The background of this study should be explained initially.**

Response to comment 2

We have added the following text to the abstract.

“The reaching motion to the back of the head with the hand is an important movement for daily living. The scores of upper limb function tests used in clinical practice alone are difficult to use as a reference when planning movement improvement exercises.”(Abstruct, lines 26–28)

**Comment 3**

**L 34, P 2: In terms of methods, it could be added that the random forest clustering analysis was conducted.**

Response to comment 3

We have added the following text to the abstract to explain the statistical analysis.

“Pattern identification using random forest clustering was performed to analyze the pattern of the motor time and the joint angle.” (Abstract, lines 36–38)

**Comment 4**

**Introduction**

**L 45, P 2: In many cases, kinematic analysis involves comparing conditions. However, this study aimed to establish kinematic cutoff values and motion patterns for the upper limb using random forest clustering. The discussion includes the application of these findings in clinical practice. The Introduction would outline the novelty of this approach and explores its clinical implications.**

Response to comment 4

We have added the following statement regarding the clinical significance of this study.

“Besides the occiput, the mouth, chest, abdomen, and lower back are other reaching target points for eating, dressing, and bathing. While important for self-care performance, reaching these target points is less difficult than reaching the occipital area, thus, patients with mild hemiplegia are often able to perform these reaches adequately [7,8]. The ability to smoothly reach the back of the head, which is difficult for hemiplegics, is beneficial to their ADLs.” (Introduction, lines 64–69)

“Due to a ceiling effect, upper extremity function scores used in clinical practice do not measure the recovery in patients with mild motor paralysis [14,15]. When planning a change in joint movement pattern exercises, or to shorten their duration, attempting to use the clinically obtained scores is challenging. To the best of our knowledge, a kinematic index that quantitatively expresses the "reach to the back of the head," does not exist. Moreover, a clear target value to determine if the reach has been sufficiently recovered in patients with mild motor paralysis is lacking.” (Introduction, lines 81–87)

“In addition, clustering patterns of movement time and joint angle changes from the data obtained using opportunity learning would be useful for providing appropriate exercises for patients [29].” (Introduction, lines 114–116)

**Comment 5**

**L 75, P 4: Please replace "Three-dimensional motion analysis" with "3DMA" for improved readability.**

Response to comment 5

We have replaced "three-dimensional motion analysis" with "3DMA" at all the related instances.

“Three-dimensional motion analysis (3DMA) is used for analyzing upper limb movement characteristics in patients with stroke [16]. Studies have been conducted to analyze the joint motion and motion time of patients with stroke using 3DMA [17,18].” (Introduction, lines 88–90)

“This issue can be analyzed using 3DMA to provide quantified data.” (Introduction, lines 99–100)

“Upper extremity motor tasks can be analyzed using 3DMA by comparing healthy subjects and patients; however, in clinical situations, movements on the paralyzed and nonparalyzed side of the patient may be compared [26].” (Introduction, lines 103–105)

“3DMA is clinically useful in patients with mild hemiparesia because the motion of the non-paralyzed upper limb is referenced, and the joint motion patterns of the paralyzed upper limb can be used to devise isolation exercises to be practiced [22]. ” (Introduction, lines 112–114)

“Based on the above discussion, in this study, we aimed to clarify the kinematic characteristics of patients with chronic phase mild hemiplegia while reaching the occipital region with the hand using 3DMA.” (Introduction, lines 117–119)

“The reaching task was analyzed using an optical 3DMA (Vicon motion system; Oxford, UK).” (Materials and methods, Data acquisition and analysis, lines 226–227)

“This is a treatment in which patients are given exercises while alternative proprioceptive feedback is returned to them based on the results measured by 3DMA and electromyography.” (Discussion, lines 531–533)

“Sixth, the cutoff values for motion time and joint angle were calculated based on the results measured using 3DMA. However, these cutoff values should be used as reference data because measurement and analysis using 3DMA is complicated and unlikely to be performed in clinical practice”. (Discussion, lines 531–533)

**Comment 6**

**L 76 – 77, P 4: Describing the system seems unnecessary.**

Response to comment 6

We have removed the following text from this manuscript.

“In this method, markers are attached to landmarks on the body and upper limb movements are recorded using an infrared camera and analyzed.”

**Comment 7**

**L 82, P 4: What does "the tasks" refer to?**

Response to comment 7

By "the tasks" we mean tasks related to daily life. We have corrected this sentence.

“Kinematic characteristics calculated by analyzing movements related to daily are strongly related to the patient's activity capacity, and are valid indicators of patient treatment in clinical practice [21–24] ” (Introduction, lines 93–96)

**Comment 8**

**L 89, P 4: If possible, consider dividing this paragraph into two sections to enhance readability.**

Response to comment 8

Thank you for your suggestion. We have divided this section into two parts.

**Comment 9**

**L 94, P 4: Delete the sentence "In a recent study…" to avoid redundancy with the following sentence, as the inertial sensors used were 3DMA.** **"In a recent study... "**

Response to comment 9

We have removed the following text from this manuscript.

“In a recent study, accelerometers, gyroscopic sensors, and magnetic sensors were attached to the bodies of patients with stroke.”

**Comment 10**

**L 102, P 5: Comparing paralyzed and non-paralyzed upper limbs is crucial for the clinical perspective of this study, which is its primary aim.**

Response to comment 10

Thank you for bringing to our attention an important point in this study. We should have been cautious in our discussion about comparing the paralyzed side with the non-paralyzed side. We have revised and added the following text in reference to your comments.

“Upper extremity motor tasks can be analyzed using 3DMA by comparing healthy subjects and patients; however, in clinical situations, movements on the paralyzed and nonparalyzed side of the patient may be compared [26]. The movements of the nonparalytic upper limb in patients with hemiplegia after a stroke differ from those of normal participants, and the reference for a paralyzed movement is not necessarily the corresponding movement of the nonparalyzed limb. Although the nonparalyzed upper limb may also have compensatory strategies for movement, a comparison of the kinematic characteristics of the paralyzed and nonparalyzed upper limbs of patients with hemiplegia with mild impairment of limb and trunk function has been conducted to validate the calculation of the evaluation values that will be used as a reference for treatment [27,28]. 3DMA is clinically useful in patients with mild hemiparesia because the motion of the non-paralyzed upper limb is referenced, and the joint motion patterns of the paralyzed upper limb can be used to devise isolation exercises to be practiced [22].” (Introduction, lines 103–114)

“Third, in the present analysis, it was not proven that the within-subject variability (left-right difference) was smaller than the between-subject variability. Because normal subjects have clearly asymmetric motor strategies in many movements, the criterion for a paralyzed movement is not necessarily the corresponding movement of the nonparalyzed limb. To resolve this limitation, the present upper limb task should be analyzed in the movements of healthy subjects to verify that it is symmetrical [66].” (Discussion, lines 620–626)

Comment 11

**Materials and methods**

**L 120, P 5: “Patients with post-stroke hemiplegia who attended the Department of…” Are they outpatients?**

Response to comment 11

The participants in our study were outpatients. We have revised the text in this regard.

“We included outpatients aged ≥20 years with a history if at least 6 months of post-stroke hemiplegia who attended the Department of Rehabilitation Medicine of the Jikei University Hospital between May 1 to October 30, 2020.” (Materials and methods, Participants, lines 136–138)

**Comment 12**

**L 130 – 131, P 6: "Higher brain dysfunction," this term is developed and commonly used only in Japan. For instance, you may rephrase it to "cognitive impairment after a stroke."**

Response to comment 12

Thank you for your valuable feedback. We have revised the text in this regard.

“The non-inclusion criteria were as follows: cases with a paralyzed hand not reaching the external occipital ridge with automatic movements; presence of a central nervous system disease other than stroke, orthopedic disease, mental disorder, cognitive impairment after stroke, dementia, visual field disorder, and ataxia at diagnosis; subluxation of the shoulder joint; pain in the joints of the upper limb or fingers during exercise; presence of a significant limitation in the joint range of motion in the upper limb; and completion of the occupational therapy intervention.” (Materials and methods, Participants, lines 143–149)

“One patient was diagnosed with cognitive impairment after stroke, two had ataxia, two had shoulder subluxation, and seven completed the occupational therapy intervention.” (Results, Participants, lines 367–369)

“due to cognitive impairment after stroke (n=1)” (Figure 4)

**Comment 13**

**L 145, P 6: “the sample size,” is this Hedge’s d? the sample size"**

Response to comment 13

The Cohen's d was used for the effect size. We have added this point to the text.

“For calculating the required sample size, the effect size (Cohen’s d) was 0.4, α was 0.05, power (1-β) was 0.8, and constant proportion was 0.5.” (Materials and methods, Sample size, lines 160–161)

**Comment 14**

**L 154, P 7: Please provide the reference for "the Plug-in Gait marker model" here.**

Response to comment 14

We have provided references in this text.

“The examiner affixed infrared reflective markers at 35 points on the participant’s body according to the Plug-in Gait marker model (Fig 3) [32].” (Materials and methods, Experimental procedure, lines 187–188)

“Reference #32 Limited VMS. Plug-in Gait reference guide. 2021 [accessed 2021 December 1]. Available from: https://docs.vicon.com/display/Nexus212/PDF+downloads+for+Vicon+Nexus”

**Comment 15**

**L 154 - 157, P 7: "The examiner made the participants sit… …and their upper limbs drooped." I did not understand what postures were set for. Is there marker setting or task measurement?** **The examiner made the participants sit... ...and their upper limbs drooped."**

Response to comment 15

We moved the description of the starting position from the Reaching task section to the Experimental procedure and modified the text accordingly.

“The examiner made the participants sit on a backless chair. The starting position of the upper limb on the measurement side was set with the elbow in full extension, fingers in extension, and the forearm in mid-position between supination and pronation; the forearm and fingers were not in contact with the chair. The upper limb on the non-tested side was also placed in the same position (Fig 1). If the starting position was difficult to achieve owing to paralysis and muscle tone, the participants were reminded to relax the muscles of the upper limbs and fingers, and the upper limbs were allowed to droop as much as possible.” (Materials and methods, Experimental procedure, lines 168–174)

**Comment 16**

**L 161, P 7: What is "a landmark" used for?**

Response to comment 16

We lacked a description of the purpose for which the landmark was placed. We have added a sentence in this regard.

“The landmark was placed to minimize neck and trunk movement while the participants performed the reaching task looking at it.” (Materials and methods, Experimental procedure, lines 179–180)

**Comment 17**

**L 164, P 7: Captions for figures must include a detailed description. For example, the position of the circles indicates the infrared markers attached to the participants’ bodies to construct the Plug-in Gait model.**

Response to comment 17

We have added the following text to the figure captions.

“Fig 3. Attachment positions of the markers. (a) Frontal image and (b) rear image. The circled positions indicate infrared markers, which were attached to 35 points on the participants' bodies to construct the Plug-in Gait model.” (Materials and methods, Experimental procedure, lines 199–201)

**Comment 18**

**L 175, P 8: Does the arrow and circle in the illustration indicate that the participants maintained their viewpoints on the landmark while performing the task?**

Response to comment 18

The viewpoints and landmarks in the figure indicate, as you pointed out, that the participants maintain their viewpoints when measuring the task. The purpose for which the landmarks were placed was added in the text (Response to comment 16). The examiner provided the following verbal instructions to all participants: “maintain the head, neck, and trunk as still as possible while looking at the landmarks placed in front during the measurement.” (Materials and methods, Reaching task, lines 205–209)

“The landmark was placed to minimize neck and trunk movement while the participants performed the reaching task looking at it.” (Materials and methods, Experimental procedure, lines 179–180)

**Comment 19**

**L 157 – 162, P 7: This section explains the setting of the starting measurements. The descriptions of the marker setting above and below this section are divided.**

Response to comment 19

Thank you for your important remarks. We have consolidated the explanations regarding the markers.

“The examiner affixed infrared reflective markers at 35 points on the participant’s body according to the Plug-in Gait marker model (Fig 3) [32]. Infrared reflective markers were applied by a single examiner in all the participants following a similar procedure. On the head, they were placed at four bilateral anterolateral and posterolateral points; on the trunk, they were placed at five points on the upper sternal body, lower sternal spine, seventh cervical vertebra, 10th thoracic vertebra, and right posterior back; and on the pelvis, at four points on the bilateral anterior and posterior superior iliac spines. On the upper limbs, they were affixed at 10 points on the bilateral upper acromion, lateral olecranon, radial and ulnar eminence, and the head of the second metacarpal. On the lower limbs, they were affixed at 12 points on the bilateral femur, lateral patella, tibia, calcaneus, external calcaneus, and head of the second metatarsal bone.” (Materials and methods, Experimental procedure, lines 187–197)

**Comment 20**

**L 179, P 8: “forearm in mid-extension.” Is this correct? forearm in mid-extension"**

Response to comment 20

There was an error in English in this text. We have corrected it. We also added a Figure showing the starting limb position during the measurement.

“The examiner made the participants sit on a backless chair. The starting position of the upper limb on the measurement side was set with the elbow in full extension, fingers in extension, and the forearm in mid-position between supination and pronation; the forearm and fingers were not in contact with the chair.” (Materials and methods, Reaching task, lines 168–171)

“Fig 1. Starting position during the measurement. (a) side view of the starting position and (b) starting position of the upper limb.” (Materials and methods, Reaching task, lines 182–183)

**Comment 21**

**L 217, P 9 – L 224, P 10: Were the peak values picked up first? Was the data for the angles, angular velocities, and displacements average of five times?**

Response to comment 21

The peak values were first extracted from 5 measurements (a total of 50 data) on 10 participants. Subsequently, descriptive data (mean, standard deviation) for angle, angular velocity, and displacement were calculated for the outward and return paths separately. We have added this point in the text.

“First, the Peak values were extracted from data obtained from 10 participants who received 5 measurements each on one upper limb (50 data in total). Next, the mean, standard deviation, maximum and minimum values of the joint angles angular velocities of shoulder flexion and abduction and elbow flexion, and displacements of the sternal pattern and occipital markers were calculated separately for outward and return motions.” (Materials and methods, Data acquisition and analysis, lines 246–251)

**Comment 22**

**L 224–225, P 10: Were the peak values of the joint angles and angular velocities?**

Response to comment 22

The peak values were present because they were first extracted from the data 50. We have added a note about this procedure in the text. (Response to comment # 21)

**Comment 23**

**L 281 – 282, P 12: “The dependent variable” and “the independent variables.” Please check whether these words must be exchanged.**

Response to comment 23

We have corrected the error as follows.

“The dependent variables were motor time, maximum values of joint angles, and angular velocities of shoulder flexion and abduction and elbow flexion, and the fixed factor was the measured side of the reaching task.” (Materials and methods, Statistical analysis, lines 308–310)

**Comment 24**

**L 288, P 12: Please explain why the cutoff values were calculated.**

Response to comment 24

We have added the following text to the Introduction to explain this point.

“The cutoff values calculated from the measured data and the clustered patterns of the movement time and joint angle changes can be used as target values in clinical practice and can be used to plan clinical practice.” (Introduction, lines 119–121)

**Comment 25**

**Results**

**L 327 and 338, P 14: Consider merging subheadings "Participants" and "Descriptive data" into a single subheading titled "Participants.**

Response to comment 25

We removed the "Descriptive data" subheading from the document and merged it into the "Participants" subheading.

**Comment 26**

**L 342 and 343, P 14: Review the necessity of including the phrase "of FMA-UE."** **of FMA-UE "**

Response to comment 26

There was an error in this sentence. We have corrected it as follows.

“The right hand was dominant in 10 participants before the onset of the stroke and switched to the left hand in 5 out of 10 participants after the onset.” (Results, Participants, lines 377–379)

**Comment 27**

**L 355, P 17 and L 368, P 20: Merge subheadings "Outcome data" and "Detection of motion features" into a unified subheading, such as "Detection of paralyzed upper limb with motion data."**

Response to comment 27

We deleted the subheadings "Outcome data" and "Detection of motion features" in the text and revised them to "Detection of paralyzed upper limb with motion data."

**Comment 28**

**L 359, P 18 and L 360, P 19: In Tables 2 and 3, examine the effect size of the Para and Non-para sides comparison using MANCOVA. The sample size (Hedges’ g = 0.4) was chosen to detect large effects.**

Response to comment 28

We analyzed the data and calculated the effect sizes. We have described our results in the text and in Tables 2 and 3.

“Table 2. Outward motion of the reaching task: results of multivariate analysis of covariance.” (Results, Detection of paralyzed upper limb with motion data)

“Table 3. Return motion of the reaching task: results of multivariate analysis of covariance.” (Results, Detection of paralyzed upper limb with motion data)

“The paralyzed side had a significantly longer motor time (F = 136.6, *p* < 0.001, η^2^ = 0.51). The peak shoulder flexion angle was significantly greater on the paralyzed side (F = 15.3, *p* < 0.001, η^2^ = 0.07), and the peak elbow flexion angle was significantly greater on the non-paralyzed side (F = 19.5, *p* < 0.001, η^2^ = 0.16). There were no differences in the peak shoulder abduction angle (F = 0.3, *p* = 0.560, η^2^ = 0.07) and peak angular velocities of shoulder flexion (F = 1.4, *p* = 0.237, η^2^ = 0.00), shoulder abduction (F = 0.9, *p* = 0.339, η^2^ = 0.00), and elbow flexion (F = 2.9, *p* = 0.090, η^2^ = 0.03; Table 2). For the return motion of the reaching task, Wilks' lambda test showed a significant main effect (F [7, 86] = 31.5, *p* < 0.001) on the measured side. Motor time (F = 98.4, *p* < 0.001, η^2^ = 0.43) was significantly longer on the paralyzed side. The peak angular velocities of shoulder flexion (F = 7.4, p = 0.008, η2 = 0.07), shoulder abduction (F = 13.9, p < 0.001, η2 = 0.12), and elbow flexion (F = 9.4, p = 0.003, η2 = 0.09) were significantly greater on the paralyzed side. The peak elbow flexion angle was significantly greater on the non-paralyzed side (F = 19.0, p < 0.001, η2 = 0.15). There was no significant difference in the peak shoulder flexion angle (F = 3.7, *p* = 0.057, η^2^ = 0.02) or peak shoulder abduction angle (F = 3.2, p = 0.075, η2 = 0.03; Table 3).” (Results, Detection of paralyzed upper limb with motion data, lines 405–419)

**Comment 29**

**L 367, P 20: Consider omitting the subheading "Main findings" to enhance readability.**

Response to comment 29

We have removed the "Main findings" subheading from the document.

**Comment 30**

**L 394 and 398, P 24: Clarify whether the probability of a non-paralyzed limb is 0. Provide an explanation on interpreting Figs 6 and 7.**

Response to comment 30

The vertical axis of the figure shows the probability of the paralyzed upper extremity; if it is 0.00, it suggests that the patient is nonparalyzed. However, it is inappropriate to state that 0.00 is the non**-**paralyzed side. We have, therefore, added an addendum to the figure caption regarding this point.

“The vertical axis of the figure is probability, and the closer the value is to 1.00, the higher the probability that the upper limb is on the paralyzed side. If the peak shoulder flexion angle was 60 degrees on the outward motion, the probability that the measured upper limb was on the paralyzed side was approximately 75%.” (Results, Detection of paralyzed upper limb with motion data, lines 432–435)

“The vertical axis of the figure is the probability, and the closer the value is to 1.00, the higher the probability that the upper limb is on the paralyzed side. If the motion time was 2.0 seconds on the return motion, the measured upper limb had approximately 80% probability of being on the paralyzed side.” (Results, Detection of paralyzed upper limb with motion data, lines 440–443)

**Comment 31**

**L 422 – 426, P 26: If feasible, elaborate on the features of the clusters focused on. Readers may find it challenging to interpret these graphs alone (Figs 9 and 10).**

Response to comment 31

We have added an explanation in the text regarding cluster 4 on the paralyzed side in the outward motion.

“For example, cluster 4 on the paralyzed side in the outward motion is characterized by longer motor time and greater angular change in shoulder flexion and abduction than the other clusters (Fig. 10).” (Results, Pattern analysis of upper limb movements, lines 468–470)

**Comment 32**

**Discussion**

**L 467 – 470, P 28: The results for the peak angle of elbow flexion are missing. Please verify them.**

Response to comment 32

Thank you for your important remarks. We have revised the manuscript in this regard.

“In contrast, the peak elbow flexion angle was significantly greater on the non-paralyzed side.” (Discussion, lines 517–518)

“As a result, during the outward reaching motion, the motor time, peak angle of shoulder flexion, and elbow flexion were calculated to be 1.6 s, 55°, and 145°, respectively.” (Discussion, lines 523–525)

**Comment 33**

**L 475 – 477, P 28: The result of the returning motion is also incorrect.**

Response to comment 33

We have modified the text as follows.

“During the return reaching motion, the motor time was significantly longer on the paralyzed side, and the peak angular velocities of elbow flexion and shoulder flexion and abduction, were significantly greater on the paralyzed side.” (Discussion, lines 515–517)

“During the return reaching motion, the motor time, peak elbow flexion angle, peak angular velocities of shoulder abduction, and elbow flexion were calculated to be 1.6 s, 145°, 53°/s, and 62°/s, respectively.” (Discussion, lines 525–527)

“The peak angular velocities of shoulder flexion (F = 7.4, *p* = 0.008, η^2^ = 0.07), shoulder abduction (F = 13.9, *p* < 0.001, η^2^ = 0.12), and elbow flexion (F = 9.4, *p* = 0.003, η^2^ = 0.09) were significantly greater on the paralyzed side. The peak elbow flexion angle was significantly greater on the non-paralyzed side (F = 19.0, *p* < 0.001, η^2^ = 0.15).” (Results, Detection of the paralyzed upper limb with motion data, lines 414–417)

**Comment 34**

**L 480, P 29: Your calculated cutoff values and motion features may serve as the target values for treating reaching occiput. Treating with these kinematic values might enhance the quality of upper limb movements (Prange‑Lasonder et al., J NeuroEngineering Rehabil, 2021, 18:162). Therefore, what improvements in movement changes can be observed in patients? Express these clinical implications and emphasize the need for future studies, as discussed in the Introduction.**

Response to comment 34

Thank you for your helpful comments. We have read the paper provided by you and have added the following text regarding the significance of this study and the application of the results.

“Kinematic quantification of movement is strongly recommended to distinguish between the recovery of motor strategies and improvement owing to motor compensations; therefore, it is increasingly used in clinical practice as a new outcome measure [19,20]. Kinematic characteristics calculated by analyzing movements related to daily life are strongly related to the patient's activity capacity, and are valid indicators of patient treatment in clinical practice [21–24]. ”(Intoroduction, lines 91–96)

“Reference # 19 Kwakkel G, Lannin NA, Borschmann K, English C, Ali M, Churilov L, Saposnik G, Winstein C, van Wegen EEH, Wolf SL, Krakauer JW, Bernhardt J: Standardized Measurement of Sensorimotor Recovery in Stroke Trials: Consensus-Based Core Recommendations from the Stroke Recovery and Rehabilitation Roundtable. Neurorehabil Neural Repair 2017;31:784-792.

“Reference #20 Demers M, Levin MF: Do Activity Level Outcome Measures Commonly Used in Neurological Practice Assess Upper-Limb Movement Quality? Neurorehabil Neural Repair 2017;31:623-637.”

“Reference #21 Alt Murphy M, Willén C, Sunnerhagen KS: Movement kinematics during a drinking task are associated with the activity capacity level after stroke. Neurorehabil Neural Repair 2012;26:1106-1115.”

“The quality of upper extremity movement can be improved by exercises performed based on kinematic values [51]. Recently, kinematic biofeedback has been developed as a new rehabilitation therapy [52]. This is a treatment in which patients are given exercises while alternative proprioceptive feedback is returned to them based on the results measured by 3DMA and electromyography. Although biofeedback has been introduced to restore the function of reaching movements in stroke patients, the effectiveness of the treatment has not been fully tested, and solid recommendations have not been provided [53]. The results of this study are useful as reference data for the application of biofeedback, robotics rehabilitation, and other technologies, and are expected to contribute to the development of therapies for restoring the patients' functions and activity limitations.” (Discussion, lines 529–538)

“Reference #51 Prange-Lasonder GB, Alt Murphy M, Lamers I, Hughes AM, Buurke JH, Feys P, Keller T, Klamroth-Marganska V, Tarkka IM, Timmermans A, Burridge JH: European evidence-based recommendations for clinical assessment of upper limb in neurorehabilitation (CAULIN): data synthesis from systematic reviews, clinical practice guidelines and expert consensus. J Neuroeng Rehabil 2021;18:162.”

“Reference #52 Giggins OM, Persson UM, Caulfield B: Biofeedback in rehabilitation. J Neuroeng Rehabil 2013;10:60.”

“Reference #53 Pelton T, van Vliet P, Hollands K: Interventions for improving coordination of reach to grasp following stroke: a systematic review. Int J Evid Based Healthc 2012;10:89-102.”

**Comment 35**

**L 485, P 29: What does "Part D" refer to?**

Response to comment 35

We have revised the text in this regard.

“Previous studies have reported that patients with stroke have a longer motor time, and the most difficult subitem in the FMA-UE is the motor time measured in Part D [54,55]. In Part D of the FMA-UE, the subject is asked to flex the elbow joint and touch the nose with the index finger five times from the position of shoulder abduction and elbow extension, which is a movement task that requires joint motion coordination as well as reaching to the back of the head.” (Discussion, lines 540–545)

**Comment 36**

**L 496, P 29: In this study, you calculated angular velocity, not angular acceleration.**

Response to comment 36

We have corrected the word error, as you noted.

“The cutoff values for the angular velocity of the joints were detected in the return reaching motion.” (Discussion, lines 556–557)

**Comment 37**

**L 541, P 31: The cut-off values proposed by this study were calculated at 0.1 second and 1 degree. These values were measured using 3DMA. However, this method is not commonly employed clinically. This point should be addressed in the Limitations section.**

Response to comment 37

Thank you for pointing out this important matter. We have added the following statement as a limitation of our study.

“Sixth, the cutoff values for motion time and joint angle were calculated based on the results measured using 3DMA. However, these cutoff values should be used as reference data because measurement and analysis using 3DMA is complicated and unlikely to be performed in clinical practice”. (Discussion, lines 632–635)

We appreciate all the comments and suggestions provided in order to improve our manuscript and we sincerely thank all the reviewers.
